# Can the Salivary Microbiome Predict Cardiovascular Diseases? Lessons Learned From the Qatari Population

**DOI:** 10.3389/fmicb.2021.772736

**Published:** 2021-12-10

**Authors:** Selvasankar Murugesan, Mohammed Elanbari, Dhinoth Kumar Bangarusamy, Annalisa Terranegra, Souhaila Al Khodor

**Affiliations:** ^1^Mother and Child Health Department, Sidra Medicine, Doha, Qatar; ^2^Clinical Research Center Department, Sidra Medicine, Doha, Qatar

**Keywords:** CVD, salivary microbiome, precision medicine, machine learning, QGP

## Abstract

**Background:** Many studies have linked dysbiosis of the gut microbiome to the development of cardiovascular diseases (CVD). However, studies assessing the association between the salivary microbiome and CVD risk on a large cohort remain sparse. This study aims to identify whether a predictive salivary microbiome signature is associated with a high risk of developing CVD in the Qatari population.

**Methods:** Saliva samples from 2,974 Qatar Genome Project (QGP) participants were collected from Qatar Biobank (QBB). Based on the CVD score, subjects were classified into low-risk (LR < 10) (*n* = 2491), moderate-risk (MR = 10–20) (*n* = 320) and high-risk (HR > 30) (*n* = 163). To assess the salivary microbiome (SM) composition, 16S-rDNA libraries were sequenced and analyzed using QIIME-pipeline. Machine Learning (ML) strategies were used to identify SM-based predictors of CVD risk.

**Results:** *Firmicutes* and *Bacteroidetes* were the predominant phyla among all the subjects included. Linear Discriminant Analysis Effect Size (LEfSe) analysis revealed that *Clostridiaceae* and *Capnocytophaga* were the most significantly abundant genera in the LR group, while *Lactobacillus* and *Rothia* were significantly abundant in the HR group. ML based prediction models revealed that *Desulfobulbus, Prevotella*, and *Tissierellaceae* were the common predictors of increased risk to CVD.

**Conclusion:** This study identified significant differences in the SM composition in HR and LR CVD subjects. This is the first study to apply ML-based prediction modeling using the SM to predict CVD in an Arab population. More studies are required to better understand the mechanisms of how those microbes contribute to CVD.

## Graphical Abstract

Salivary microbiome marker selection: CVD risk score using ML model in the Qatari population.

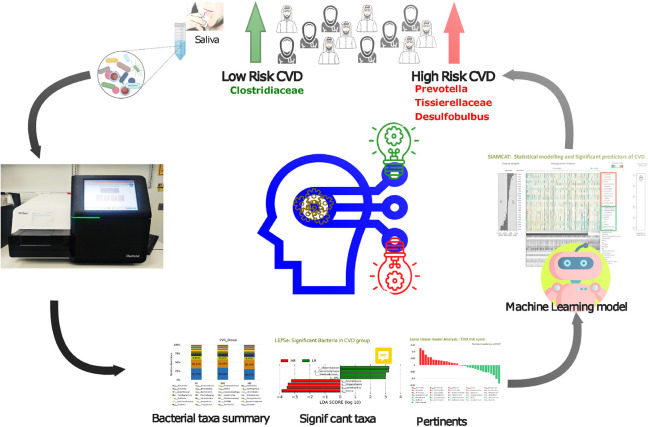

## Introduction

Non-communicable Diseases (NCDs) are the leading cause of death globally (Allen et al., 2017). According to the World Health Organization [WHO] (2013) report, the global burden of non-communicable diseases (NCDs) raised to 82% by 2020. The most common NCDs are cardiovascular diseases (CVD), cancer, respiratory disorders, and diabetes (Balakumar et al., 2016).

CVD comprises coronary heart disease, heart failure, stroke, rheumatic heart disease, and cardiomyopathies among others (Caldwell et al., 2019). CVD is the leading cause of death, claiming about 17.9 million deaths annually and increasing worldwide (Lear et al., 2017; Al-Shamsi et al., 2019).

In Qatar, NCDs are the leading cause of death for the past 10 years (Al-Kaabi and Atherton, 2015) with the CVD mortality rates reaching 8.3 per 100000 MOPH (2020). In addition, the 2006-World-Health-Survey revealed that the Qatari population suffers from various predisposing factors to CVD such as obesity (28.8%), high cholesterol (24.7%), diabetes (16.7%), and hypertension (14.4%) Haj Bakri and Al-Thani (2012).

In the past decade, advances in the multi-omics technologies have enhanced our chances to discover novel biomarkers (Olivier et al., 2019). Blood-based biomarkers are considered invasive, there is an urgent need to use non-invasive samples such as saliva to develop new disease biomarkers. In addition, the advance in Next-Generation Sequencing platforms (NGS) has enabled us to assess the human microbiome with an unprecedented resolution and depth. Using the human microbiome composition to identify disease biomarkers is the next chapter of precision medicine (Morganti et al., 2019; Zhong et al., 2021).

The human microbiome (HM) comprises trillions of bacteria, viruses, protozoa, and fungi that reside in and on our body surfaces (Amon and Sanderson, 2017). The HM is complex, dynamic, ubiquitous, and shows striking variability from one individual to another and between various body sites (Ursell et al., 2012; Aagaard et al., 2013). The HM has a wide array of roles ranging from digestion, protection from pathogens, immune-regulation, and metabolites production (Marchesi et al., 2016). The oral cavity harbors more than 700 diverse microorganisms and is considered the second most diverse site after the gut (Deo and Deshmukh, 2019). In healthy subjects, the core salivary microbiome (SM) includes genera *Streptococcus, Veillonella, Neisseria*, and *Actinomyces* (Zaura et al., 2009, 2014). In a large-scale population-based Japanese study, the authors showed that the SM is dominated by *Streptococcus, Neisseria, Rothia, Prevotella, Actinomyces, Granulicatella, Haemophilus*, and *Porphyromonas* (Yamashita and Takeshita, 2017). Our previous study aiming to characterize the salivary microbiome composition in the Qatari population (Murugesan et al., 2020) showed that *Bacteroidetes, Firmicutes, Actinobacteria*, and *Proteobacteria* were the common phyla, with *Bacteroidetes* being the most predominant (Murugesan et al., 2020). Dysbiosis in the SM is associated with oral diseases (Mashima et al., 2017; Davis et al., 2020) and systemic diseases like obesity, diabetes, and CVD (Wade, 2013; Kholy et al., 2015; Cortez et al., 2019).

Advances in Machine Learning (ML) technologies, an essential branch of artificial intelligence, have enabled researchers to build prediction biomarker models for various diseases such as arthritis, diabetes, and inflammatory bowel disease (Jamshidi et al., 2019; Aryal et al., 2020; Kohli et al., 2020). On the other hand, few studies have trained ML models using the gut microbiome profiles to identify predictors of atherosclerosis and CVD (Aryal et al., 2020; Liu et al., 2020) and none have used the SM so far.

This study aims to identify whether a predictive salivary microbiome signature is associated with a high risk of developing CVD in the Qatari population. We integrated the phenotypic, clinical, and microbiome data, and we identified SM-biomarkers associated with an increased risk to CVD using ML models.

## Materials and Methods

### Ethics Statement

The study was approved by the Institutional Review Board (IRB) of Sidra Medicine under (protocol #1510001907) and by Qatar Biobank (QBB) (protocol #E/2018/QBB-RES-ACC-0063/0022. All study participants signed an informed consent before sample collection. All experiments were performed under the approved guidelines.

### Clinical Data

We collected de-identified saliva samples, phenotypic and clinical data from a total of 2,974 participants enrolled in the Qatar genome project (QGP). QGP included any adult who is either a Qatari national or long-term resident (lived in Qatar for at least 15 years) and can contribute to QBB around 3 h of their time for answering all the questionnaires, complete measurements, imaging and fitness assessments, in addition to providing all the samples required including saliva. In the pilot phase, the cohort consisted of 1,432 males and 1,542 females (Table 1). Each subject’s anthropometric and blood parameters were established by analyzing body mass index (BMI), total protein content, hemoglobin, albumin, ferritin, calcium, iron, vitamin-D, high or low-density lipoprotein cholesterol (HDL, LDL), triglycerides, and glucose levels.

**TABLE 1:** Clinical parameters of the study cohort.

	LR (*N* = 2491)	MR (*N* = 320)	HR (*N* = 163)	*P*-value
Male (*N* = 1432)	1184	161	87	<0.001^a^***
Female (*N* = 1542)	1307	159	76	<0.001^a^***
CVD score	2.78 ± 2.48	13.89 ± 2.75	31.76 ± 11.87	<0.001^b^***
BMI	28.37 ± 5.86	30.51 ± 4.76	31.18 ± 5.80	<0.001^b^***
Age	35.11 ± 10.22	50.89 ± 7.15	55.87 ± 8.14	<0.001^b^***
APT	33.82 ± 2.97	33.82 ± 2.97	33.13 ± 3.05	0.011^b^*
Albumin (gm/L)	44.30 ± 3.31	44.16 ± 3.16	43.14 ± 3.59	0.001^b^**
Alkaline phosphatase (U/L)	70.02 ± 20.66	75.71 ± 21.32	76.39 ± 21.70	<0.001^b^***
ALT (GPT) (U/L)	22.02 ± 16.54	28.67 ± 16.15	27.72 ± 15.11	<0.001^b^***
AST (GOT) (U/L)	19.89 ± 16.80	21.08 ± 7.83	20.39 ± 7.41	<0.001^b^***
Calcium (mmol/L)	2.29 ± 0.08	2.30 ± 0.095	2.32 ± 0.10	<0.001^b^***
Cholesterol total (mmol/L)	4.92 ± 0.93	5.37 ± 1.11	5.44 ± 1.28	<0.001^b^***
C-Peptide (ng/mL)	2.14 ± 1.30	2.88 ± 2.22	2.83 ± 1.38	<0.001^b^***
Creatinine (μmol/L)	65.24 ± 13.90	74.04 ± 13.91	77.71 ± 19.86	<0.001^b^***
Dihydroxy VitD Total (ng/mL)	17.65 ± 11.46	19.57 ± 11.35	19.13 ± 9.43	<0.001^b^***
Ferritin (mcg/L)	65.02 ± 105.93	109.76 ± 96.33	124.33 ± 101.1	<0.001^b^***
Fibrinogen (gm/L)	3.29 ± 0.68	3.40 ± 0.65	3.48 ± 0.67	0.001^b^**
Folate (nmol/L)	20.64 ± 7.51	22.42 ± 7.25	22.82 ± 7.44	<0.001^b^***
Free thyroxine (pmol/L)	12.96 ± 1.89	12.73 ± 1.85	12.82 ± 1.46	0.006^b^**
Glucose (mmol/L)	5.18 ± 1.50	6.71 ± 2.91	7.92 ± 3.79	<0.001^b^***
HbA1C	5.40 ± 0.83	6.28 ± 1.56	7.14 ± 1.95	<0.001^b^***
HDL-Cholesterol (mmol/L)	1.43 ± 0.38	1.19 ± 0.30	1.12 ± 0.29	<0.001^b^***
Hemoglobin (gm/dL)	13.44 ± 1.79	14.59 ± 1.44	14.45 ± 1.56	<0.001^b^***
Insulin (mcunit/mL)	12.31 ± 14.90	19.03 ± 27.04	16.25 ± 12.89	<0.001^b^***
INR	1.05 ± 0.09	1.01 ± 0.09	1.00 ± 0.10	<0.001^b^***
Iron (μmol/L)	14.92 ± 6.71	16.59 ± 5.75	16.18 ± 5.74	<0.001^b^***
LDL-Cholesterol (mmol/L)	2.96 ± 0.87	3.29 ± 1.20	3.37 ± 1.18	<0.001^b^***
Potassium (mmol/L)	4.36 ± 0.37	4.44 ± 0.38	4.51 ± 0.42	<0.001^b^***
Total protein (gm/L)	73.67 ± 3.90	73.26 ± 3.82	73.15 ± 3.81	0.083^b^
Triglyceride (mmol/L)	1.16 ± 0.69	1.81 ± 1.18	1.94 ± 1.15	<0.00^b^***
Urea (mmol/L)	4.21 ± 1.25	4.75 ± 1.21	5.07 ± 1.84	<0.001^b^***

*APT, activated partial thromboplastin time; BMI, body mass index; INR, International Normalization Ration, PT, prothrombin time; TSH, thyroid stimulating Hormone; TIBC, total iron binding capacity.*

*^a^Chi-square test, ^b^Kruskal–Wallis test.*

*P-value < 0.05, **P-value < 0.01, ***P-value < 0.001.

### Calculation of Cardiovascular Diseases Risk Score

Cox proportional-hazards regression has been used to evaluate the risk of developing CVD over 10-years. The CVD-risk score for 2974 patients was estimated using sex-specific multivariable factors consisting of age, total-Cholesterol, HDL, systolic blood pressure (BP), hypertension treatment, smoking, and diabetes status (HbA1C ≥ 6.5%, and participants who confirmed having diabetes). D’Agostino et al. (2013) adapted the regression coefficient for the functions from earlier analysis. This method uses the following equation:


$$\hat{p}=1-S_{0}\,{\left(t\right)}^{\exp\left(\sum_{i=1}^{p}\beta_{i}\,x_{i}-\sum_{i=1}^{p}\beta_{i}\,{\bar{x}}_{i}\right)}$$

Where S_0_(t), baseline survival at follow-up time t (here *t* = 10 years); β_*i*_, estimated regression coefficient (log hazard ratio that is measured for all risk functions and sex-specific); x_*i*_, log-transformed value of the ith risk factor; i, corresponding mean, p, number of risk factors.

#### Sample Collection

Qatar Biobank collected saliva samples according to standard procedure. They organized to collect 5 mL of spontaneous, whole, unstimulated saliva into a 50 mL sterile DNA-free Falcon tube from each participant by spitting. The samples were divided into 0.4 mL aliquots and stored at −80 C until further analysis. The aliquots were received from QBB for total salivary DNA extraction.

#### DNA Extraction and 16S rRNA Gene Sequencing

The total salivary DNA was extracted using automated QIAsymphony protocol (Qiagen, Hilden, Germany), following the Manufacturer’s instructions. DNA purity was evaluated by the A260/A280 ratio using a NanoDrop 7000 Spectrophotometer (Thermo Fisher Scientific, Waltham, MA, United States), and the DNA integrity was checked on a 1% agarose by gel electrophoresis.

The V1–V3 regions of the 16S rRNA gene were amplified using Illumina NextEra XT library preparation Kit (FC-131-1002). Step 1 PCR is performed using 10 ng of template DNA for 50 μL PCR reaction using 2X Phusion Hot Start Ready mix (Thermo Fisher Scientific™). The following thermal cycling conditions were used: 5 min of initial denaturation at 94°C; 25 cycles of denaturation at 94 C for 30 s, annealing at 55°C for 30 s, extension at 72 C for 30 s; and a final extension at 72 C for 5 min. According to the Manufacturer’s instructions, the amplified PCR products of approximately 550 bp in size was purified using AgenCourt AMPure XP magnetic beads (Beckman Coulter). Purified PCR products of STEP 1 was used as template for amplification of STEP 2 NextEra index PCR using thermocycling conditions of 5 min of initial denaturation at 94°C; 8 cycles of denaturation at 94 C for 30 s, annealing at 55°C for 30 s, extension at 72 C for 30 s; and a final extension at 72 C for 5 min. These PCR products were purified using AgenCourt AMPure XP magnetic beads and purified products were pooled in equimolar concentrations. High-throughput sequencing was performed using an Illumina MiSeq 2 × 300 platform following the manufacturer’s instructions.

### 16S rRNA Sequencing Data Analysis

Demultiplexed sequence data were revised for quality control using FastQC (Andrews, 2010). PEAR tool was used to merge both forward and reverse sequence reads of respective samples (Zhang et al., 2014), and sequence reads of quality score <20 were discarded. All merged reads were trimmed to 160 bp > Reads < 500 bp using the Trimmomatic tool (Bolger et al., 2014). Trimmed FASTQ files were converted into FASTA files. Demultiplexed FASTA files were analyzed using Quantitative Insights Into Microbial Ecology (QIIME) v1.9.0 pipeline (Caporaso et al., 2010; Murugesan et al., 2020). Operational taxonomic units (OTUs) were generated by aligning against the Greengenes database (Version: 13_8) with a confidence threshold of 97% (DeSantis et al., 2006).

### Statistical Taxonomic and Diversity Analyses

Linear Discriminant Analysis Effect Size (LEfSe) (Segata et al., 2011) was used to find differentially abundant taxa between the studied categories. Alpha diversity measures including Chao1, Observed, Shannon, and Simpson indices were calculated with R-phyloseq package (McMurdie and Holmes, 2013). The alpha diversity statistical significance was calculated using Mann–Whitney test through Minitab-17 (2010). *P*-values less than 0.05 were considered statistically significant. Differences in the beta diversity were presented as principal coordinate analysis using QIIME. Analysis of similarities (ANOSIM) was used to calculate the distance matrix difference between the categories using Bray-Curtis metric (Caporaso et al., 2010).

### Supervised Machine Learning Modeling

**FIGURE 1:**
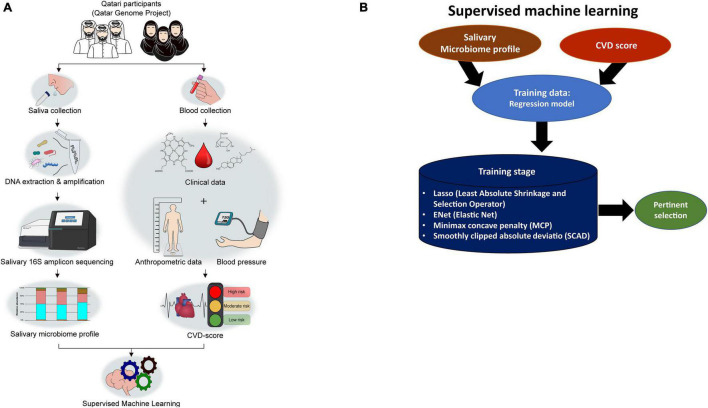
Overall study design from participant recruitment to SM-based CVD marker selection. **(A)** The study workflow. **(B)** Strategies applied in Supervised machine learning (ML) to select pertinents.

We applied four statistical ML methods for regularization and feature selection based on penalized least squares (Figure 1B). The methods are the Least Absolute Shrinkage and Selection Operator (Lasso), Smoothly Clipped Absolute Deviation Penalty (Zou and Li, 2008) (SCAD), Elastic Net (Zou and Hastie, 2005) (ENet), and Minimax concave penalty (Zhang, 2010) (MCP). The methods differ by the mathematical properties of the corresponding penalties: Lasso and ENet use convex penalties, while MCP and Scad use concave penalties. We applied two transformations to the abundance-counts as in: a binary transformation (Binary), and a variance-stability transformation (Arcsin), while the CVD-score outcome was log-transformed (Dong et al., 2020). Analyses were performed using the R-packages glmnet (Hastie and Qian, 2014) and ncvreg (Breheny, 2020). The graphics were generated using the R-packages ggplot2, RVenn, and ggpubr (Wickham, 2011; Akyol, 2019; Kassambara, 2020). We randomly split the data 50-times into a training set (80%) on which the predictive-models were build and a test-set (20%) on which we tested the performance of each model. Optimal tuning parameters were chosen *via* 10-fold cross-validation.

## Results

### Demographic and Clinical Parameters of the Study Population

The study population was composed of 2,974 Qatari participants. The cohort was classified into three CVD groups as low-risk (LR) (CVD score < 10), moderate-risk (MR) (CVD score: 10–20), and high-risk (HR) (>20), as described in the section “Materials and Methods.” As a result, 2491 participants were LR, 320 were MR, and 163 were HR (Table 1). The average participant’s age in the HR group (55.87 ± 8.14 years) was significantly higher than those in the MR (50.89 ± 7.15 years) and LR (35.11 ± 10.22 years) groups (Table 1). Moreover, the BMI was significantly higher in the HR group than in the MR and LR groups (Table 1). In addition, among the blood parameters tested, Alkaline phosphatase, Calcium, Total-Cholesterol, LDL, Creatinine, Ferritin, Fibrinogen, Folate, Glucose, HbA1C, Urea, and Triglycerides were significantly higher in the HR group (Table 1).

### The Salivary Microbiome Composition Reveals Signatures for Cardiovascular Diseases

**FIGURE 2:**
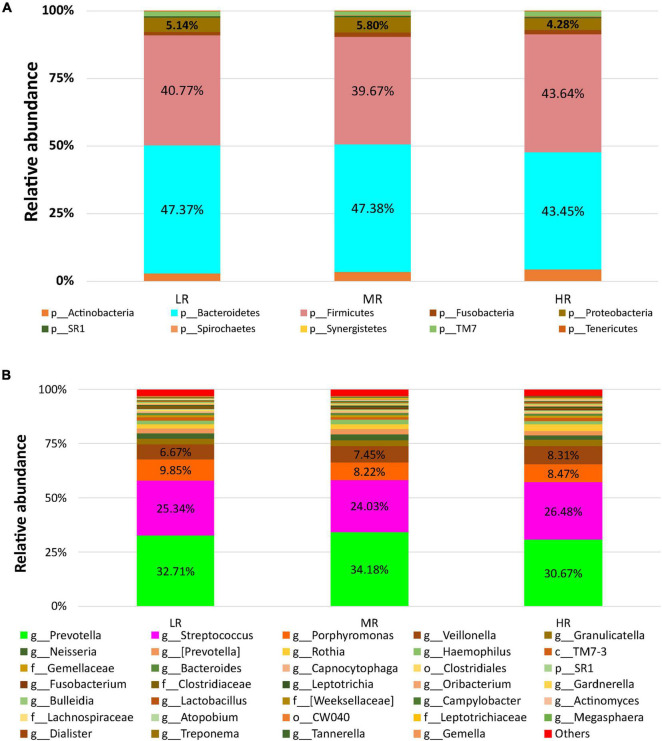
The salivary microbiome composition of CVD risk groups. *Y*-axis shows % of relative abundance of the microbiome; *X*-axis indicates the microbial abundance in LR, MR, and HR groups; **(A)** phylum level; **(B)** genus level.

After stratifying the study cohort based on the CVD risk score, we assessed the SM composition in all subjects. Then, we compared the compositional changes between different study groups. A diagram that summarizes the study design is shown in Figures 1A,B. The microbial sequence data generated from all the participants revealed 22 bacterial phyla, 46 classes, 87 orders, 173 families, and 390 genera. *Bacteroidetes, Firmicutes, Actinobacteria*, and *Proteobacteria* were the most abundant phyla observed in the saliva samples collected from the Qatari subjects, covering approximately 90% of total microbial abundance (Figure 2A). In addition, our data showed that *Streptococcus, Prevotella, Porphyromonas, Granulicatella, and Veillonella* represent the salivary core microbiome members at the genus level (Figure 2B).

### Differential Salivary Microbial Taxa Between the High-Risk and Low-Risk-Cardiovascular Diseases Groups

**FIGURE 3:**
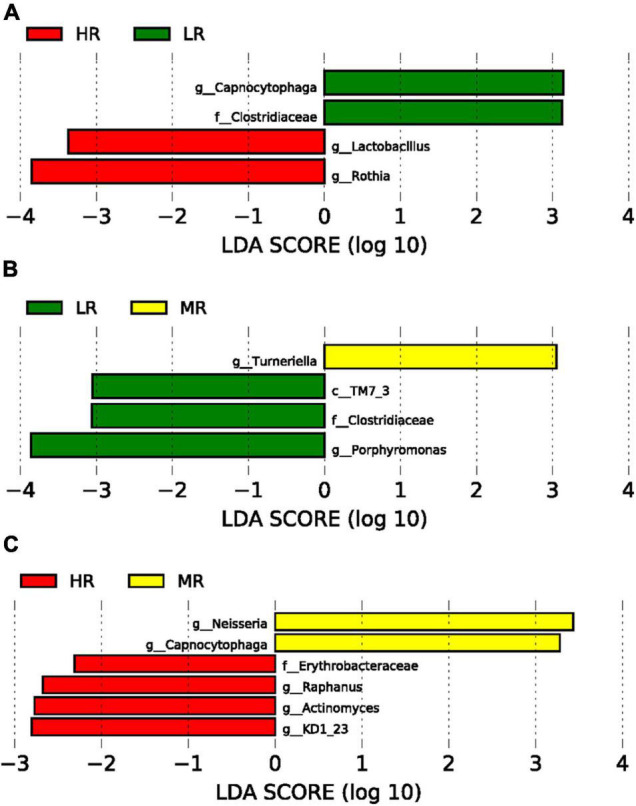
Graphs of linear discriminant analysis (LDA) scores for differentially enriched bacterial genera among the groups. **(A)** LR (green) vs. HR (red) groups; **(B)** LR (green) vs. MR (Yellow) groups; **(C)** HR (red) vs MR (Yellow) groups.

After assessing the study cohort’s SM, LEfSe analysis compared the salivary microbiome compositions in the LR, MR, and HR (Figure 3). Our data indicated that *Capnocytophaga* and *Clostridiaceae* were significantly abundant in the LR group compared to the HR group (*p* < 0.0001). In contrast, *Lactobacillus* and *Rothia* were significantly enriched in the HR group (*p* < 0.0001) (Figure 3A) in comparison to the LR group. *Clostridiaceae* and *Porphyromonas* were significantly increased in the LR group than MR group. *Neisseria* and *Capnocytophaga* were greatly enriched in the MR group than HR group (Figures 3B,C).

Alpha and beta diversity measures were calculated to assess the changes in diversity among groups (Supplementary Figure 1). Alpha diversity parameters revealed no significant differences observed between all groups (Supplementary Figure 1A). We then performed beta diversity analysis to assess the divergence in the community composition between the groups using the Bray-Curtis distance metric (Supplementary Figure 1B). We showed that the salivary microbiome in HR and MR were not significantly dissimilar from the LR group (*p* = 0.085) (Supplementary Figure 1B).

### Identification of Pertinent Salivary Microbial Markers Associated With the Cardiovascular Diseases Score Using Machine Learning Models

The apparent differences between the study groups using alpha and beta diversity measures were not identified due to the significant sample size differences and imbalance. In this study, the participants were selected from the QGP Cohort, who provided saliva samples exclusively. QBB collected the biosamples from all volunteers as a sampling of Qatari population without focusing on CVD risk-based recruitment. We decided to use regression-based ML selection of pertinent SM biomarkers to avoid bias based on the sample size. The data were split 50-times randomly, using the four feature selection techniques, and the whole dataset was used without any exclusion (Figure 1B).

**FIGURE 4:**
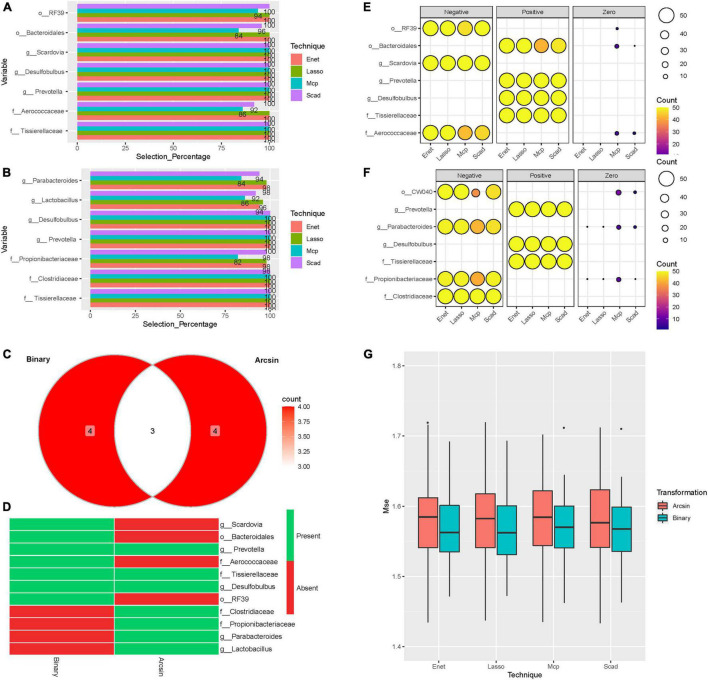
Machine learning models. Barplots representing the selection percentages of the microbes selected at least 80% of the time by the four methods over the 50 random splits of the data. **(A)** Binary transformation. **(B)** Arcsin transformation. **(C)** Venn Diagram showing the number of microbes. **(D)** Heatmap [presence (green)/absence (red)] of selected microbes using Binary and Arcsin transformations. **(E)** Balloon plot representing sign counts of the regression coefficients: Binary transformation **(F)** Arcsin transformation. The size of circles represents the number of splits. The color represents the number of counts. **(G)** Box plots of the MSE for the four-methods and the two transformations applied to the microbiome abundance data. Each point of the boxplot represents the MSE on the test-set.

To search for pertinent variables, we focused on the abundances of SM selected at least 80% of the time among the 50-random splits of the data and the four feature selection techniques as described in the section “Materials and Methods.” Our results are shown in Figure 4. Seven microbes were selected at least 80% of the time using the binary and Arcsin transformations by all the ML methods (Lasso, SCAD, ENet, and MCP) (Figures 4A,B). Three microbes were presented at all the tested models and both transformations (Figures 4C,D). In comparison, four microbes were specific to the binary transformation and four were particular to the Arcsin transformation (Figure 4D). The common microbes were *Prevotella, Tissierellaceae*, and *Desulfobulbus* (Figure 4D). To better understand how these microbes affect the CVD-score, we counted the sign of the regression coefficients number of times, Positive, Negative, or Zero (Figure 4E). From this analysis, the three microbes mentioned above contribute to an increase in the CVD score (Figure 4E). At the same time, our data showed that an increase in *Clostridiaceae* level contributed to a decrease in CVD-score (Figure 4F). Assessment using the Mean squared error (MSE) method disclosed that binary transformation has better prediction accuracy than Arcsin (Figure 4G).

## Discussion

The need for practical, non-invasive tools for predicting and preventing CVD risk has led to concerted research efforts in recent years to identify and characterize biomarkers associated with the disease as a step forward toward precision medicine. In addition, recent studies on the microbiome have enlightened its role in human health and disease (Solbiati and Frias-Lopez, 2018). Despite that, the diversity of the gut microbiome is affected by several factors like gender, ethnicity, age, and environmental factors; it was found to be associated with many diseases, including CVD and IBD using ML-models (Gulden, 2018; Chang and Kao, 2019). However, the potential use of the SM composition in assessing CVD is still lacking.

This study evaluated whether the SM composition can predict a high risk for developing CVD in a diverse Qatari population. Using a large cohort of 2,974 Qatari participants and based on the CVD risk score, we showed for the first time that the SM composition in LR and HR individuals is different (LefSe analysis). A significant SM alteration was observed between LR, MR, and HR groups (Figures 3A–C). Furthermore, *Capnocytophaga* and *Clostridiaceae* were significantly enriched in the LR group (Figure 3A). While no studies are addressing the role of *Capnocytophaga* in health and disease, a study among Japanese patients showed that non-ischemic heart failure is associated with lower levels of *Clostridiaceae* (Katsimichas et al., 2018). In line with our findings, a significant reduction of *Clostridiaceae* was observed in the HR-CVD group in the Qatari population (Figures 3A,4D,F).

Moreover, our data showed that *Lactobacillus* and *Rothia* were enriched in the HR group compared to the LR group (Figure 3A). Similarly, a study aiming to utilize the gut microbiome as a diagnostic marker of coronary artery disease (CAD) in the Japanese population has revealed that *Lactobacilli* were more abundant in patients with CAD than their matching controls (Emoto et al., 2017). On the other hand, *Rothia*, a nitrate-reducing bacterium, was enriched in hypertensive patients (Wang et al., 2021).

Next, we employed a novel approach of regression-based machine learning by combining the entire dataset of 16S rDNA sequencing data with ML models to identify the potential predictors of HR CVD without stratifying the cohort to mask the bias due to sample size differences among groups. We found that three microbes (*Prevotella, Tissierellaceae*, and *Desulfobulbus)* were represented by binary and Arcsin transformations and different training model techniques. Those were associated with high CVD-score (Figure 4). The Bogalusa Heart Study aimed to associate the lifetime CVD risk among the participants using the gut microbes revealed that the genus *Prevotella* was significantly enriched in the CVD HR participants (Kelly et al., 2016). Also, the role of gut microbiome in Chinese CVD patients with cardiac valve calcification revealed that *Prevotella* is a potential pathogen that is positively correlated with LDL (Liu et al., 2019). Moreover, hypertensive rats had a significant increase of *Tissierellaceae* in the gut microbiome (Sherman et al., 2018). Furthermore, *Tissierella soehngenia* was more abundant in rats with acute myocardial infarction than in the control groups (Wu et al., 2017). *Tissierellaceae* produces trimethyl amino N-oxide (TMAO), a known microbial metabolite associated with heart attack, stroke, and chronic kidney disease (Al-Obaide et al., 2017). Our study showed that *Desulfobulbus –* sulfidogenic bacterium (Devkota et al., 2012) has a positive regression coefficient with CVD scores in both trained models (Figures 4C,D). The elevated level of *Desulfobulbus* is known to trigger proinflammatory cytokines in patients with rheumatoid arthritis and periodontitis (Eriksson et al., 2019). Moreover, its abundance is positively correlated with age rendering it an excellent predictor to diagnose systemic diseases like diabetes and CVD (Tomas et al., 2012).

To our knowledge, this study is the first to demonstrate the promising potential of artificial intelligence *via* ML modeling for a convenient prediction screening of CVD based on the SM composition in the Arab population. While most ML strategies based on the health records (including age, sex, smoking habit, systolic BP, total cholesterol, HDL, cholesterol, BP treatment, and diabetes), fewer studies used gut microbiome profiles to predict IBD and CVD with an AUC of ≈0.70 and 0.90, respectively (Masetic and Subasi, 2016; Weng et al., 2017; Aryal et al., 2020; Tsoi et al., 2020; Manandhar et al., 2021). A pilot study of Japanese patients with atherosclerotic cardiovascular disease (ACVD) revealed that SM could be used as an optimal marker of ACVD with an AUC of 0.933 (Kato-Kogoe et al., 2021). It is a promising finding to enable the discovery of non-invasive biomarkers that can predict the risk of the disease before it occurs. This study is novel, and the outcomes will be a step toward developing new biomarkers for early non-invasive testing aiming to reduce the CVD burden. The main limitation of this study is the single time point recruitment of the participants without any follow-up on the participants, in addition to the imbalance in the sample size between the groups. This study mainly focuses on the SM shift with a change in CVD-score. In this study, we did not consider the other confounding factors such as chronic diseases like diabetes, arthritis, and hypertension and their treatment, which can also influence the SM shift.

Further studies are warranted to confirm our findings and the potential use of these microbial signatures as diagnostic or prognostic markers. In addition, more investigation of these biomarkers for their mechanistic and pathophysiological evidence could be helpful in the personalized approach to treat CVD.

## Data Availability Statement

The datasets presented in this study can be found in online repositories. The names of the repository/repositories and accession number(s) can be found below: NCBI (accession: PRJNA781451).

## Ethics Statement

The studies involving human participants were reviewed and approved by the Institutional Review Board (IRB) of Sidra Medicine under (protocol #1510001907) and Qatar Biobank (QBB) (protocol #E/2018/QBB-RES-ACC-0063/0022). The patients/participants provided their written informed consent to participate in this study.

## Author Contributions

SAK designed the study, obtained funds for the project, reviewed the data, and finalized the manuscript. SM processed the samples, analyzed the data, and wrote the initial draft. AT and DB calculated the CVD scores and reviewed the data and the manuscript. ME analyzed the data using ML techniques. All authors reviewed and accepted the final version of the manuscript.

## Conflict of Interest

The authors declare that the research was conducted in the absence of any commercial or financial relationships that could be construed as a potential conflict of interest.

## Publisher’s Note

All claims expressed in this article are solely those of the authors and do not necessarily represent those of their affiliated organizations, or those of the publisher, the editors and the reviewers. Any product that may be evaluated in this article, or claim that may be made by its manufacturer, is not guaranteed or endorsed by the publisher.

## Supplementary Material

Supplementary Figure 1 **(A)** Alpha diversity measures for the LR, MR, and HR groups. **(B)** Principal Coordinates Analysis (PCoA) based on Bray-Curtis distances of SM.
