# Supplementary figures and images for: Can the Salivary Microbiome Predict Cardiovascular Diseases? Lessons Learned From the Qatari Population
Source: Front Microbiol. 2021 Dec 10;12:772736. doi: 10.3389/fmicb.2021.772736 (PMC8703018; doi:10.3389/fmicb.2021.772736)

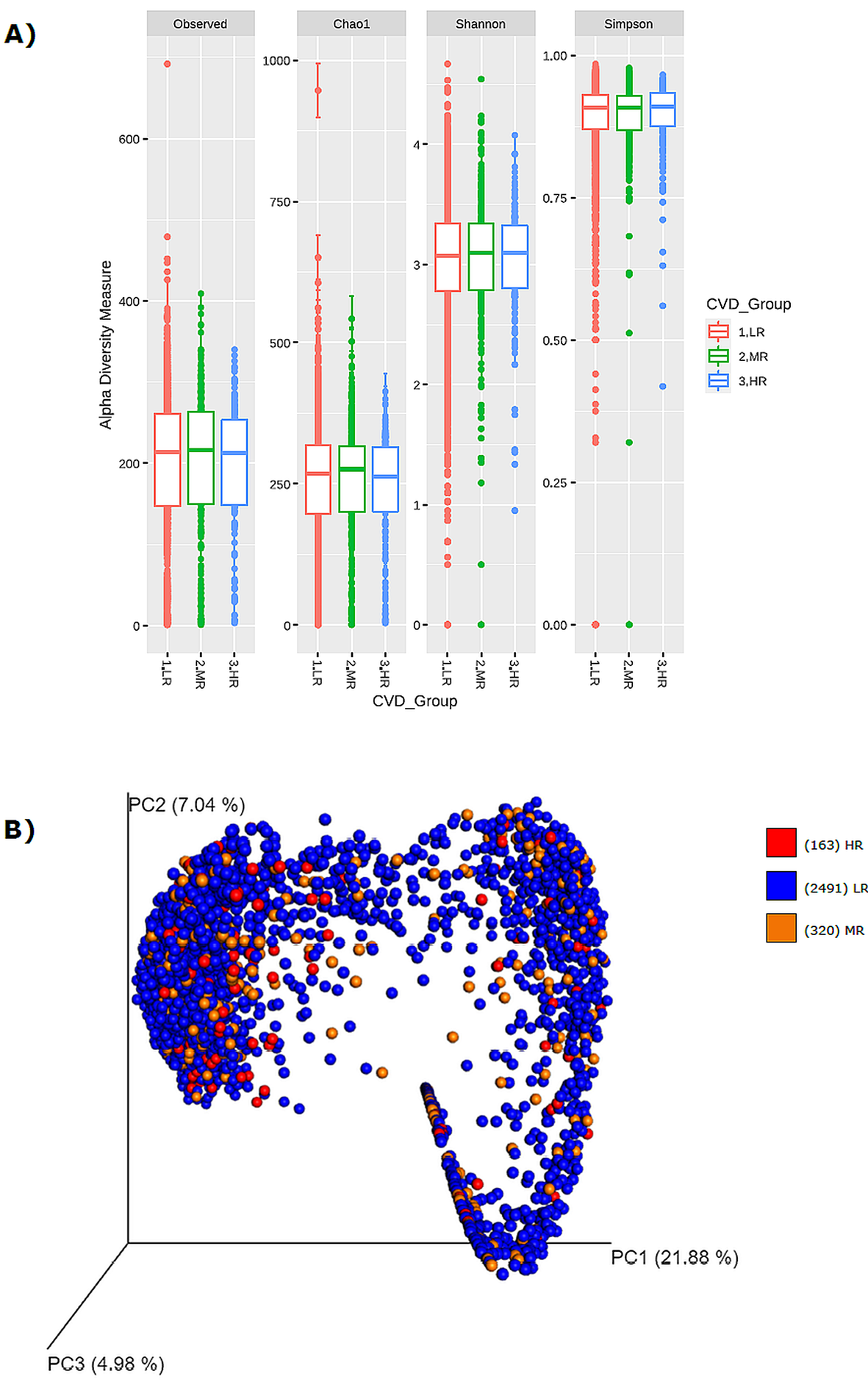

Supplement: Supplementary Figure 1 — (A) Alpha diversity measures for the LR, MR, and HR groups. (B) Principal Coordinates Analysis (PCoA) based on Bray-Curtis distances of SM. [file Image_1.TIF]
